# Supplementary figures and images for: Naa12 compensates for Naa10 in mice in the amino-terminal acetylation pathway
Source: eLife. 2021 Aug 6;10:e65952. doi: 10.7554/eLife.65952 (PMC8376253; doi:10.7554/eLife.65952)

GENE®

Nac10

— +/Y +mla/Y —

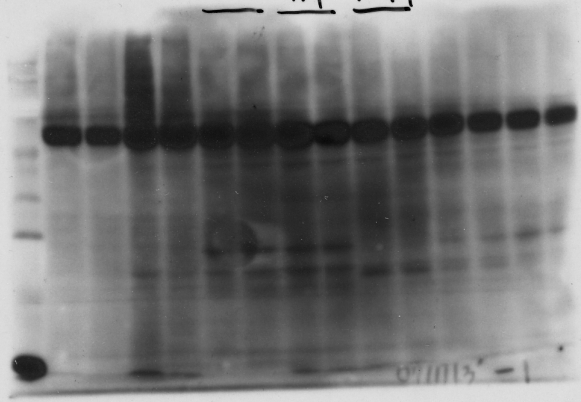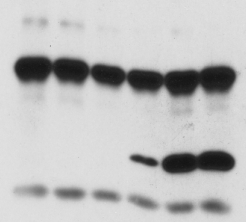

STRATA

Supplement: Figure 1—figure supplement 1—source data 1. [file elife-65952-fig1-figsupp1-data1.pdf]

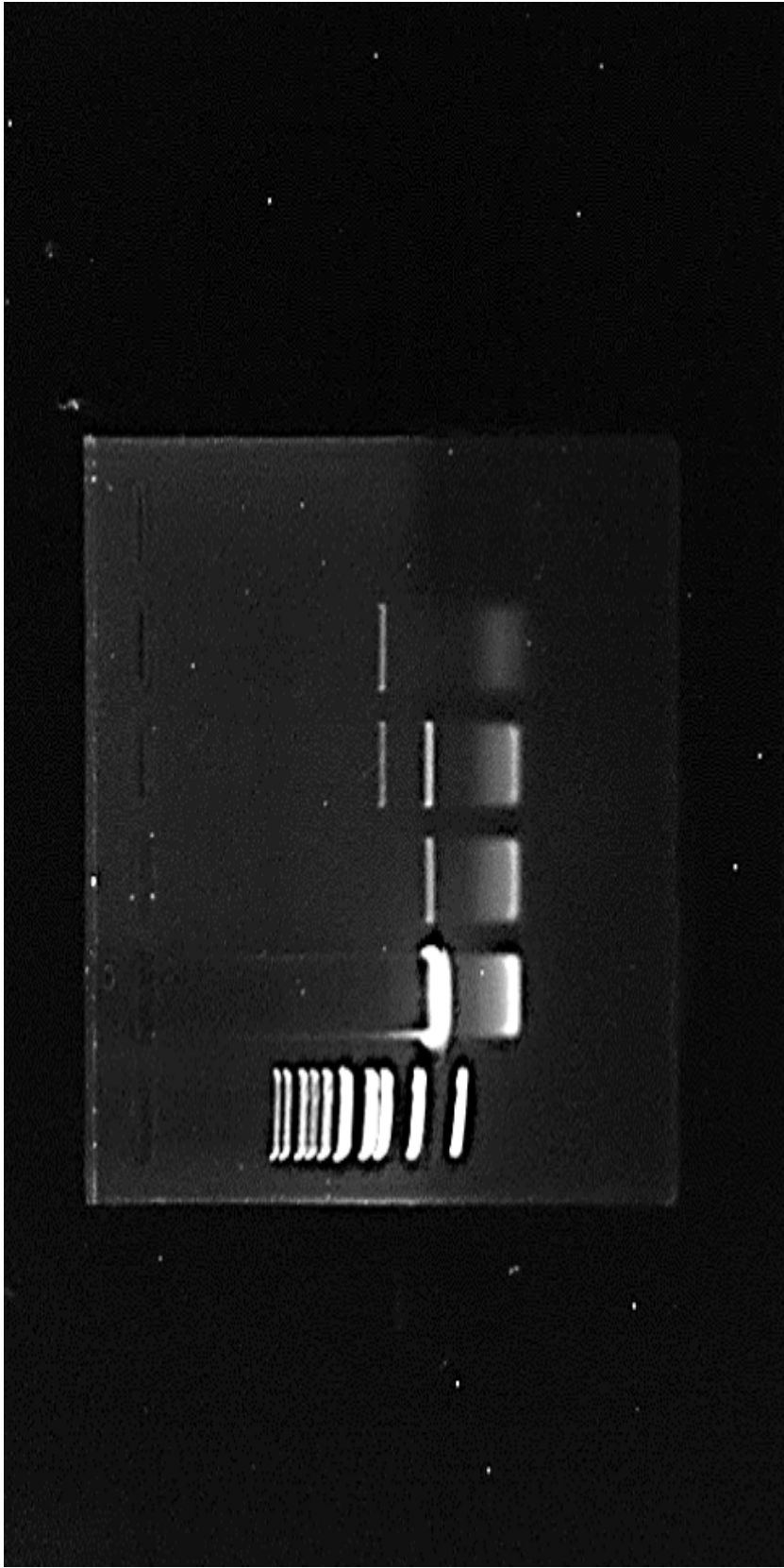

Supplement: Figure 1—figure supplement 1—source data 2. [file elife-65952-fig1-figsupp1-data2.pdf]

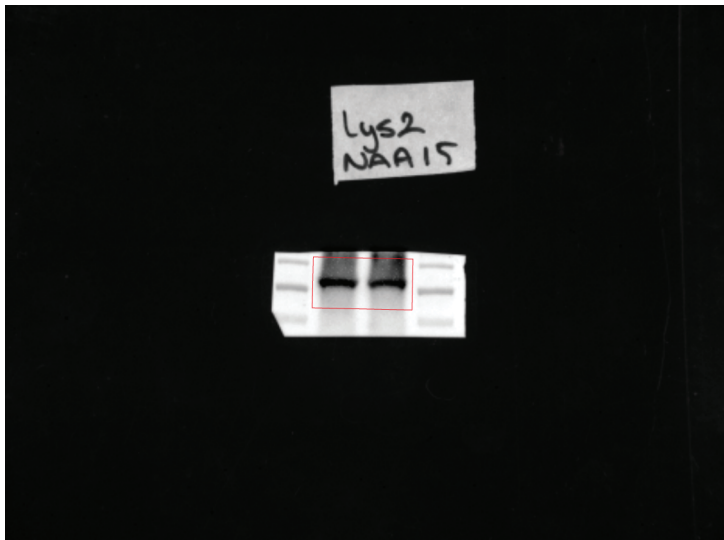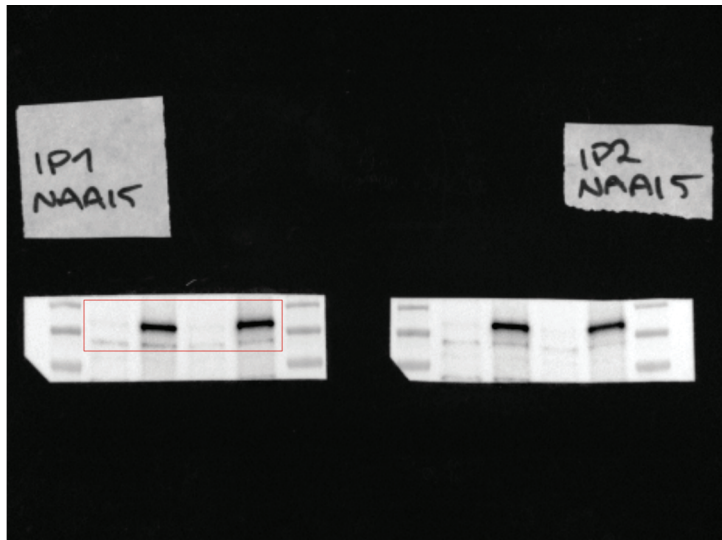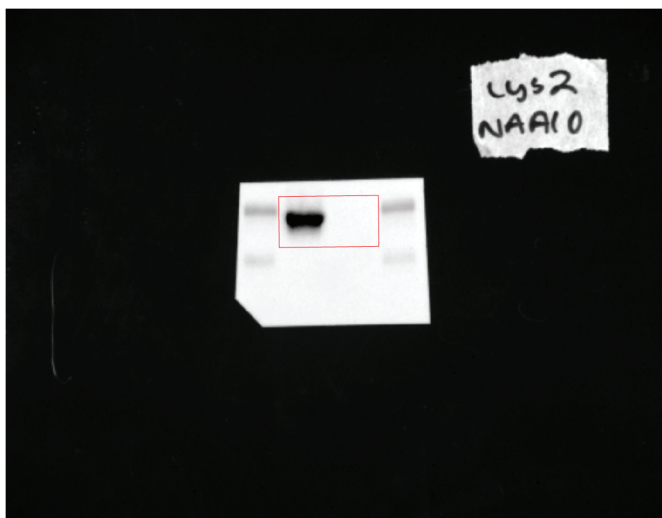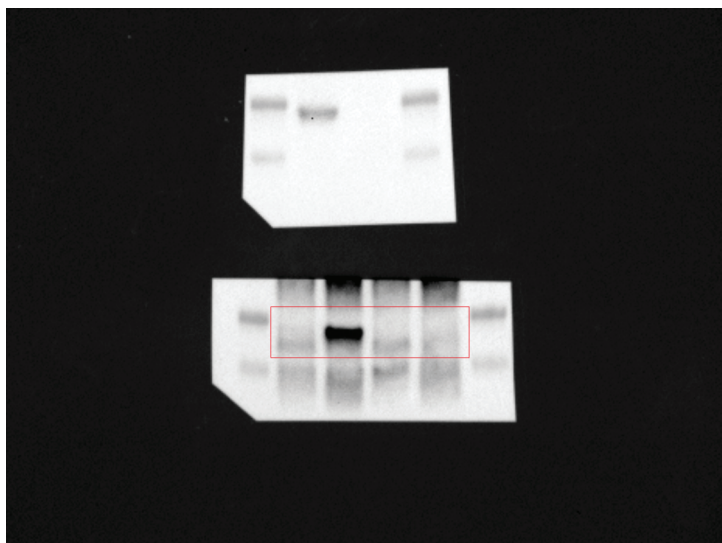

Supplement: Figure 3—source data 1. [file elife-65952-fig3-data1.pdf]

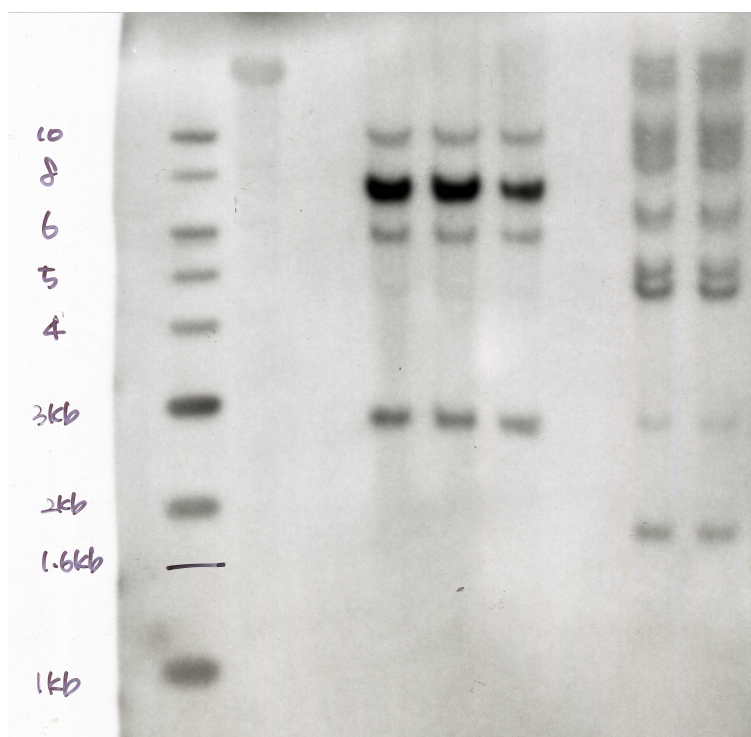

Supplement: Figure 3—figure supplement 1—source data 1. [file elife-65952-fig3-figsupp1-data1.pdf]

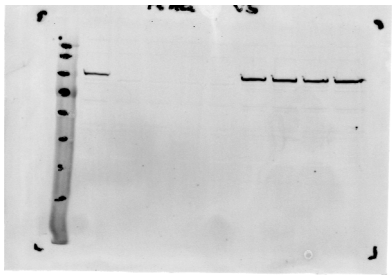

Supplement: Figure 4—source data 1. [file elife-65952-fig4-data1.pdf]

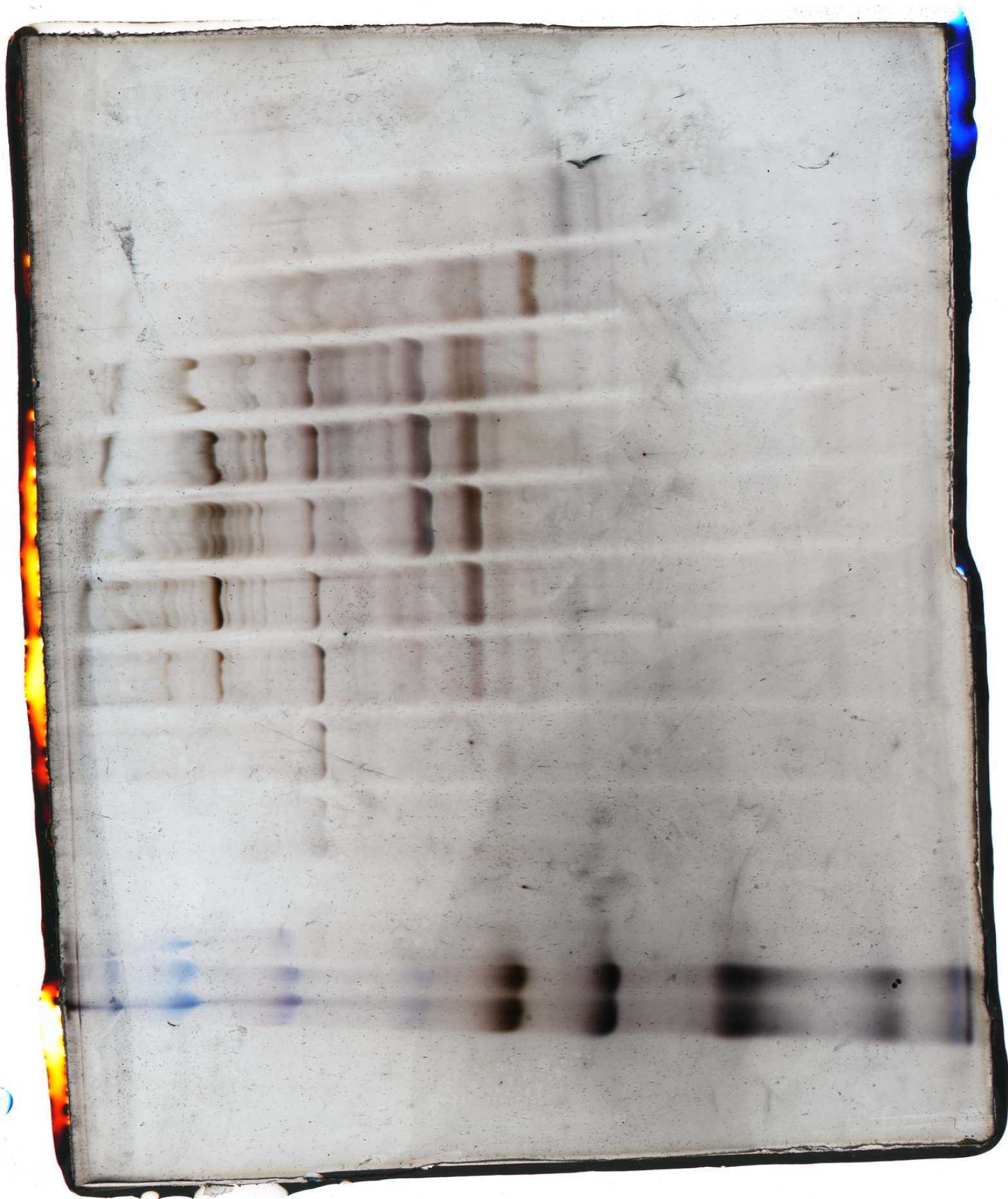

Supplement: Figure 4—figure supplement 1—source data 1. [file elife-65952-fig4-figsupp1-data1.pdf]

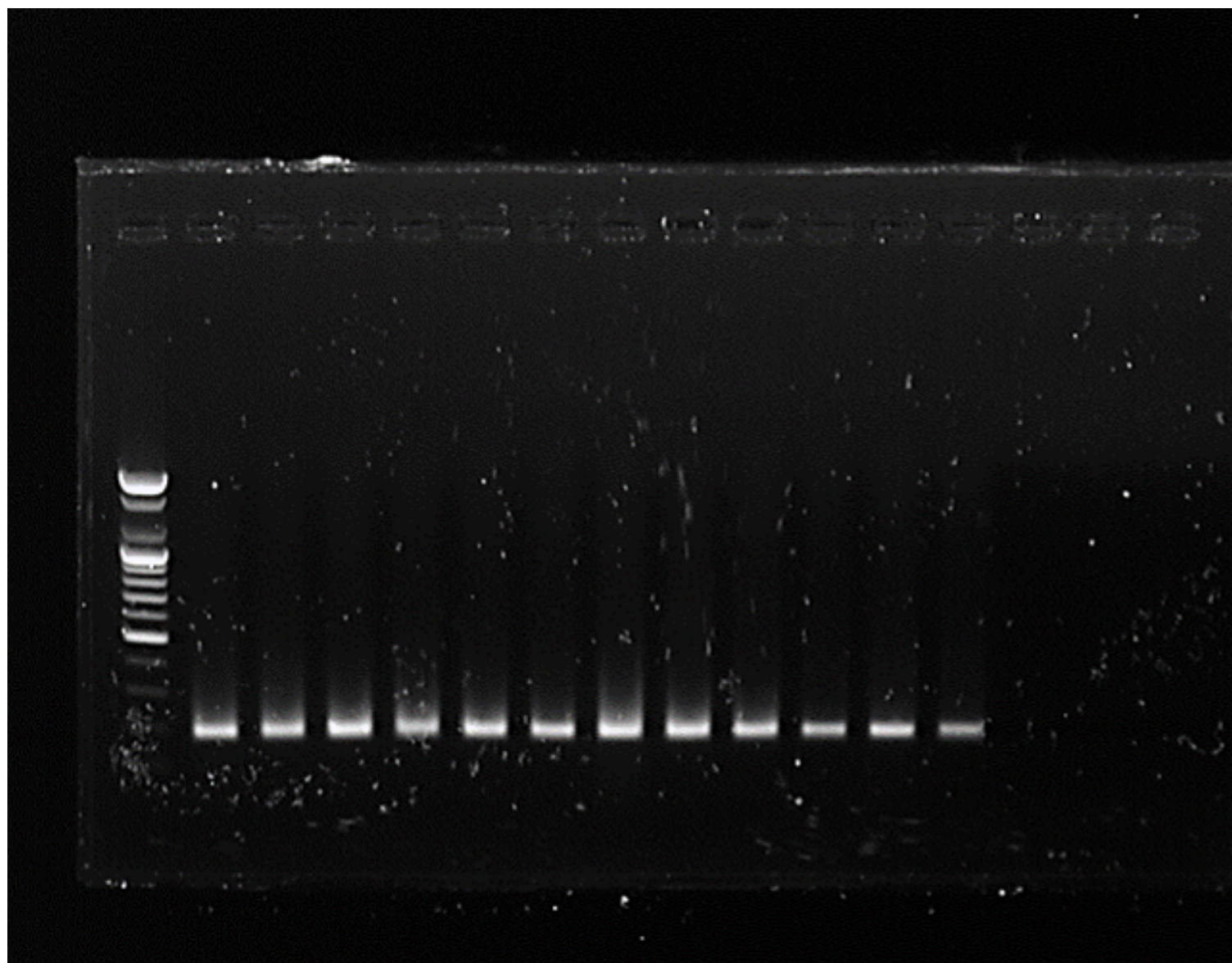

Supplement: Figure 4—figure supplement 1—source data 2. [file elife-65952-fig4-figsupp1-data2.pdf]

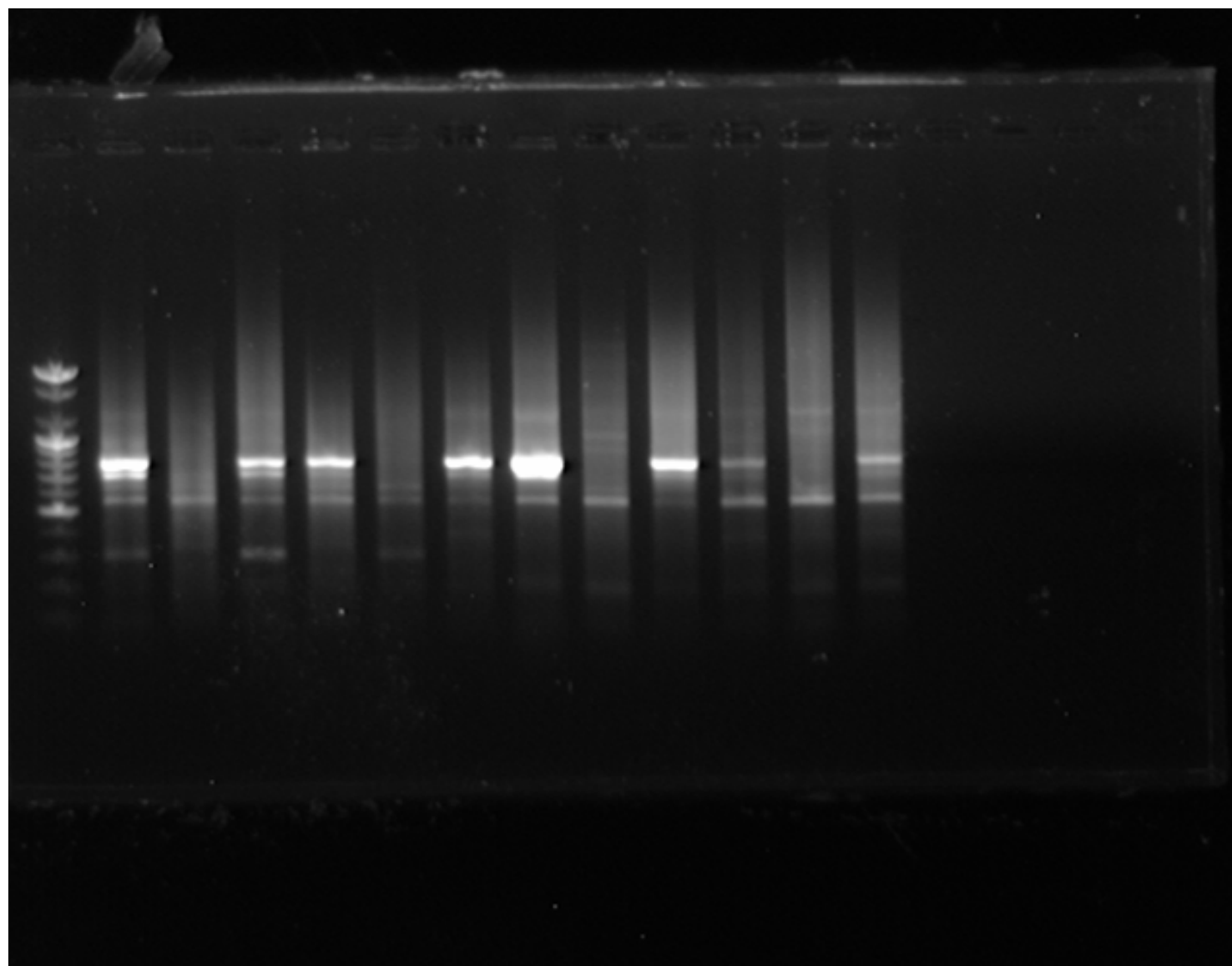

Supplement: Figure 4—figure supplement 1—source data 3. [file elife-65952-fig4-figsupp1-data3.pdf]

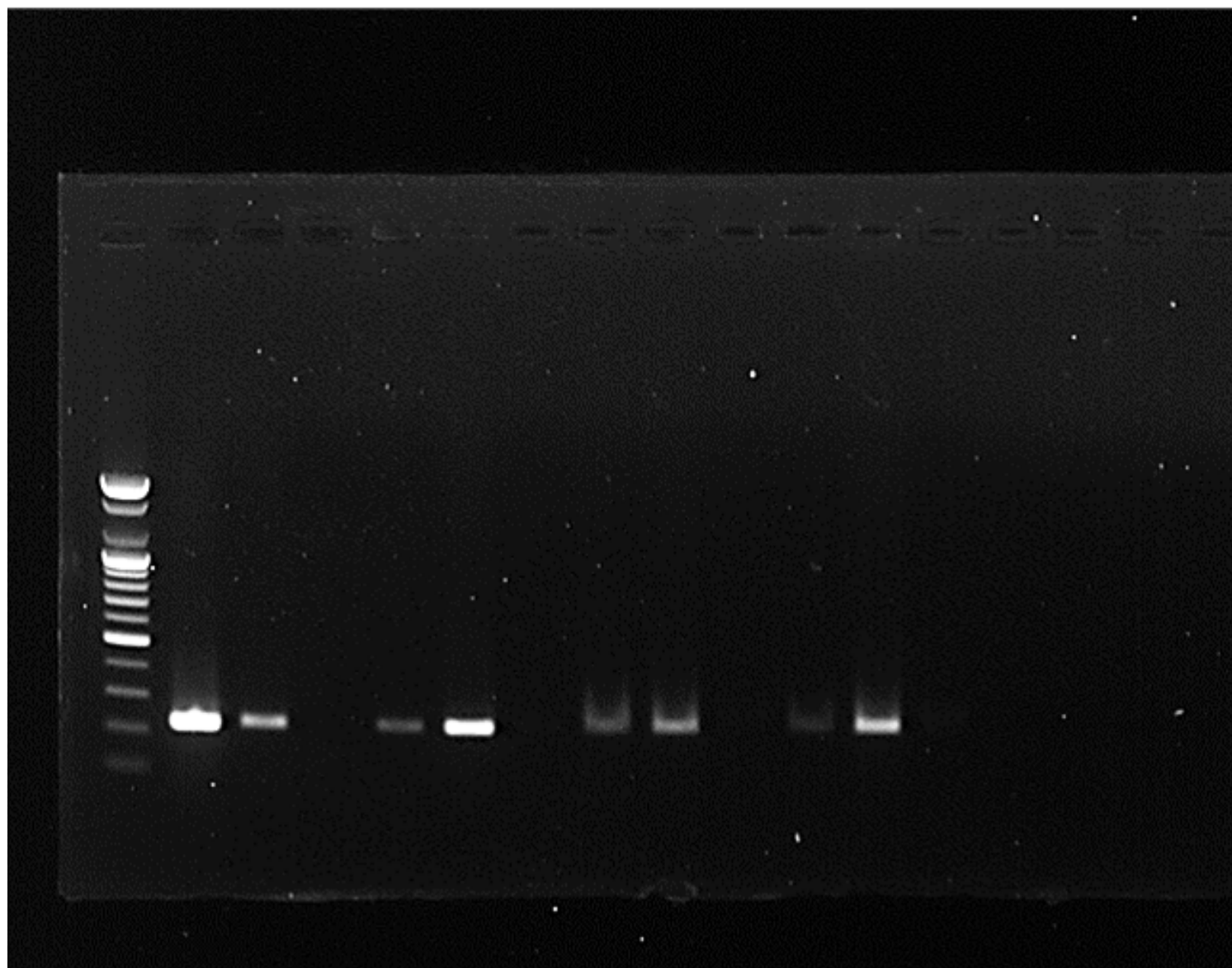

Supplement: Figure 4—figure supplement 1—source data 4. [file elife-65952-fig4-figsupp1-data4.pdf]

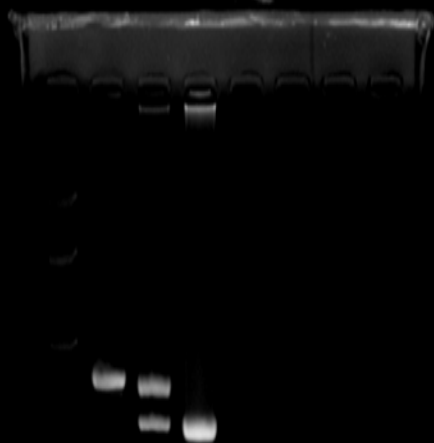

Supplement: Figure 5—source data 1. [file elife-65952-fig5-data1.pdf]
