# Supplementary material for: Naa12 compensates for Naa10 in mice in the amino-terminal acetylation pathway
Source: eLife. 2021 Aug 6;10:e65952. doi: 10.7554/eLife.65952 (PMC8376253; doi:10.7554/eLife.65952)
Supplement: Figure 1—figure supplement 1—source data 3. [file elife-65952-fig1-figsupp1-data3.pdf]

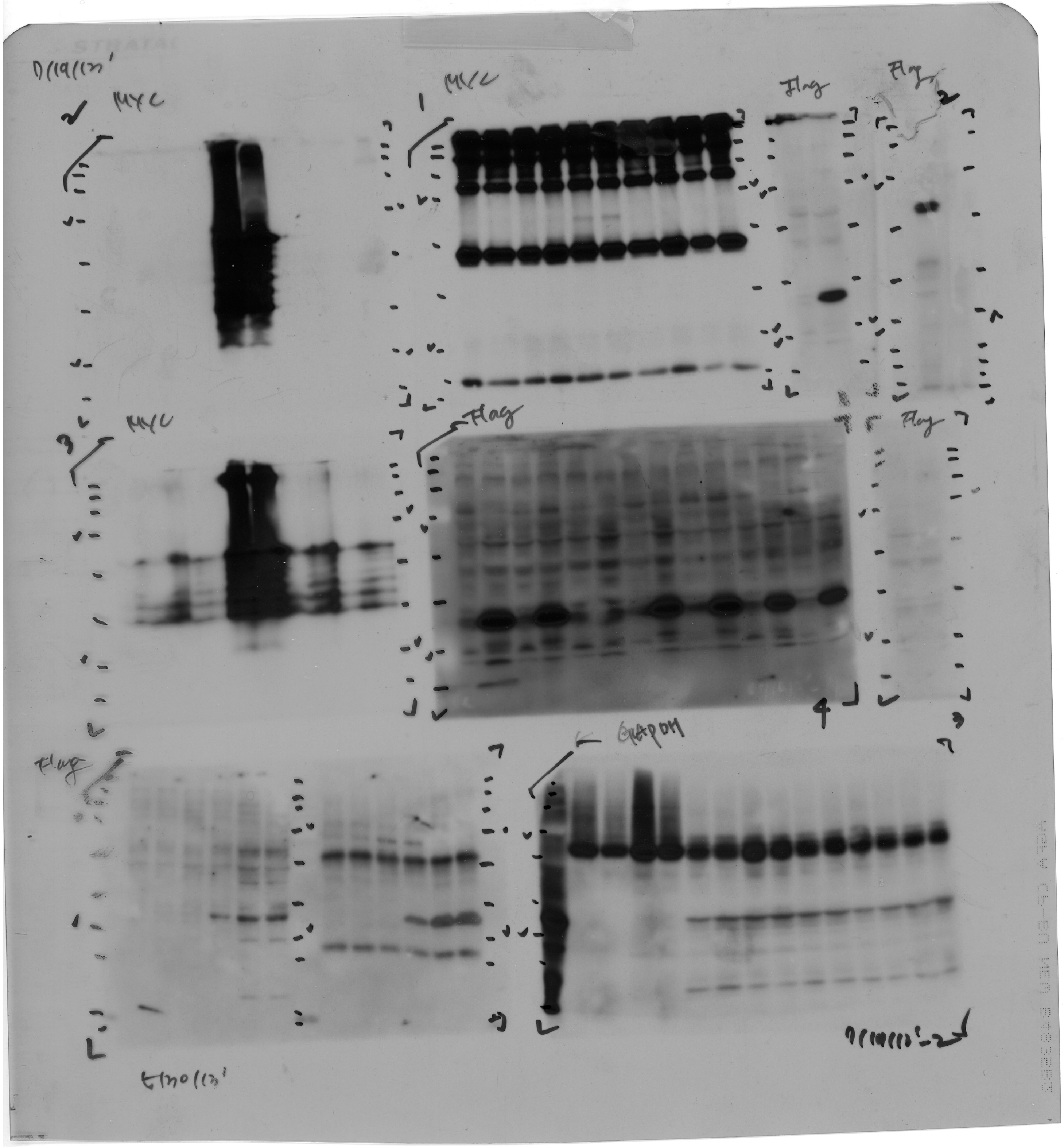
MYC
MYC
Flag
Flag
MYC
Flag
Flag
Flag
GAPDH
