# Supplementary material for: Naa12 compensates for Naa10 in mice in the amino-terminal acetylation pathway
Source: eLife. 2021 Aug 6;10:e65952. doi: 10.7554/eLife.65952 (PMC8376253; doi:10.7554/eLife.65952)
Supplement: Supplementary file 1. — (a) Genotypes of offspring from Naa10+/- female mice crossed to the Naa10+/Y male mice. Expected and observed Mendelian ratio of genotypes in offspring at E10.5, E13.5, E18.5 and adults from crosses of Naa10+/- female and Naa10+/Y male mice. The percentage of adult Naa10-/Y mice significantly decreases. (b) Genotypes of offspring from Naa10+/tm1a female mice crossed to the Naa10+/Y male mice. Expected and observed Mendelian ratio of genotypes in offspring at E10.5, E13.5, E18.5 and adults from crosses of Naa10+/tm1a female and Naa10+/Y male mice. The percentage of adults Naa10tm1a/Y mice significantly decreases. (c) Cervical fusion skeletal analyses in Naa10 knockout (KO) mice. (d) Matings and litter size analyses. (e) Genotypes of offspring from Naa12+/- female mice crossed to the Naa12+/- male mice. Expected and observed Mendelian ratio of genotypes in offspring from crosses. (f) Genotypes of offspring from Naa10+/- Naa12+/+ female mice crossed to the Naa10+/y Naa12+/- male mice. Expected and observed Mendelian ratio of genotypes in offspring from crosses. (g) Genotypes of offspring from Naa10+/- Naa12+/+ female mice crossed to the Naa10+/y Naa12-/- male mice. Expected and observed Mendelian ratio of genotypes in offspring from crosses. (h) Mendelian and observed offspring distributions from Naa10(+/Y); Naa12(+/-) male and Naa10(+/-); Naa12(+/-) female breeding. (i) Mendelian and observed offspring distributions from Naa10(+/Y); Naa12(-/-) male and Naa10(+/-); Naa12(+/-) female breeding. (j) Mendelian and observed postnatal offspring distributions from Naa10(+/Y); Naa12(+/-) male and Naa10(+/-); Naa12(+/+) female breeding. (k) Mendelian and observed postnatal offspring distributions from Naa10(+/Y); Naa12(-/-) male and Naa10(+/-); Naa12(+/+) female breeding. (l) Mendelian and observed age-specific offspring distributions from four crosses. (m) Mendelian and observed cumulative offspring distributions from all four crosses. (n) Mice analyzed by weighing, according [file elife-65952-supp1.docx]

# Supplementary File 1.

Supplementary File 1a. Genotypes of offspring from *Naa10^+/-^* female mice crossed to the *Naa10^+/Y^* male mice.

| **Genotype**  **(Expected Mendelian %)** | **Naa10^+/Y^ or Naa10^+/+^ (50%)** | **Naa10^+/-^ (25%)** | **Naa10^-/Y^ (25%)** |
| --- | --- | --- | --- |
| **E10.5 (n=134)** | **62 (46.3%)** | **39 (29.1%)** | **33 (24.6%)** |
| **E13.5 (n=98)** | **53 (54.1%)** | **22 (22.4%)** | **23 (23.4%)** |
| **E18.5 (n=170)** | **82 (48.2%)** | **49 (28.8%)** | **39 (23.0%)** |
| **Adults (n=733)** | **438 (59.8%)** | **207 (28.2%)** | **88 (12.0%)** |

Expected and observed Mendelian ratio of genotypes in offspring at E10.5, E13.5, E18.5 and adults from crosses of *Naa10^+/-^* female and *Naa10^+/Y^* male mice. The percentage of adult *Naa10^-/Y^* mice significantly decreases.

Supplementary File 1b. Genotypes of offspring from *Naa10^+/tm1a^* female mice crossed to the *Naa10^+/Y^* male mice.

| **Genotype**  **(Expected Mendelian %)** | **Naa10^+/Y^ or**  **Naa10^+/+^ (50%)** | **Naa10^+/tm1a^ (25%)** | **Naa10^tm1a/Y^ (25%)** |
| --- | --- | --- | --- |
| **E10.5 (n=109)** | **55 (50.46%)** | **26 (23.85%)** | **28 (25.69%)** |
| **E12.5 (n=45)** | **20 (44.4%)** | **12 (26.7%)** | **13 (28.9%)** |
| **E18.5 (n=53)** | **27 (51.0%)** | **13 (24.5%)** | **13 (24.5%)** |
| **Adults (n=260)** | **152 (58.5%)** | **85 (32.7%)** | **23 (08.8%)** |

Expected and observed Mendelian ratio of genotypes in offspring at E10.5, E13.5, E18.5 and adults from crosses of *Naa10^+/tm1a^* female and *Naa10^+/Y^* male mice. The percentage of adults *Naa10^tm1a/Y^* mice significantly decreases.

Supplementary File 1c. Cervical fusion skeletal analyses in *Naa10* KO mice.

| **genotype** | **sample size** | **one or more**  **fusion events** | **two or more**  **fusion events** | **consecutive**  **fusion events** | **C1+2 fusion** | **C2+3 fusion** | **C3+4 fusion** | **C4+5 fusion** | **C5+6 fusion** | **C6+7 fusion** | **C7+ T1 fusion** | **T1+2 fusion** |
| --- | --- | --- | --- | --- | --- | --- | --- | --- | --- | --- | --- | --- |
| ***Naa10^+/Y^*** | **19** | **2/17 (12%)** | **1/17 (6%)** | **0/17**  **(0%)** | **2/17 (12%)** | **0/18 (0%)** | **0/18 (0%)** | **0/19 (0%)** | **0/19 (0%)** | **0/19 (0%)** | **1/19 (5%)** | **0/19 (0%)** |
| ***Naa10^+/+^*** | **4** | **1/4 (25%)** | **0/4**  **(0%)** | **0/4**  **(0%)** | **1/4 (25%)** | **0/4**  **(0%)** | **0/4**  **(0%)** | **0/4**  **(0%)** | **0/4**  **(0%)** | **0/4**  **(0%)** | **0/4**  **(0%)** | **0/4**  **(0%)** |
| ***Naa10^+/-^*** | **4** | **1/4 (25%)** | **0/4**  **(0%)** | **0/4**  **(0%)** | **1/4 (25%)** | **0/4**  **(0%)** | **0/4**  **(0%)** | **0/4**  **(0%)** | **0/4**  **(0%)** | **0/4**  **(0%)** | **0/4**  **(0%)** | **0/4**  **(0%)** |
| ***Naa10^-/Y^*** | **9** | **9/10 (90%)** | **3/9 (33%)** | **1/9**  **(11%)** | **7/10 (70%)** | **2/9 (22%)** | **2/9 (22%)** | **1/9 (11%)** | **0/9**  **(0%)** | **0/9**  **(0%)** | **1/9 (11%)** | **0/9**  **(0%)** |
| ***Naa10^-/-^*** | **1** | **1/1 (100%)** | **1/1 (100%)** | **1/1**  **(100%)** | **1/1 (100%)** | **1/1 (100%)** | **0/1**  **(0%)** | **0/1**  **(0%)** | **0/1**  **(0%)** | **0/1**  **(0%)** | **0/1**  **(0%)** | **0/1**  **(0%)** |

Supplementary File 1d. Matings and litter size analyses.

| ***Naa10* KO matings, all WT/WT for Naa12, all >99.6% C57BL/6J** | | | | | | | **Naa10 x Naa12 matings,**  **mixed genetic background** | |
| --- | --- | --- | --- | --- | --- | --- | --- | --- |
| **Genotypes of breeders (♀ x ♂)** | ***Naa10^+/+^* x**  ***Naa10^+/Y^*** | ***Naa10^+/+^* x**  ***Naa10^-/Y^*** | ***Naa10^+/-^* x *Naa10^+/Y^*** | ***Naa10^+/-^* x**  ***Naa10^-/Y^*** | ***Naa10^-/-^* x**  ***Naa10^-/Y^*** | ***Naa10^-/-^* x *Naa10^+/Y^*** | **Naa10^+/-^ Naa12^+/+^**  **x**  **Naa10^+/Y^ Naa12^+/-^** | **Naa10^+/-^ Naa12^+/+^**  **x**  **Naa10^+/Y^ Naa12^-/-^** |
| **#pups** | **255** | **18** | **330** | **59** | **59** | **127** | **214** | **252** |
| **#litters** | **39** | **2** | **66** | **13** | **11** | **31** | **43** | **64** |
| **#pups/**  **#litters, or litter size** | **6.5** | **9.0** | **5.0** | **4.5** | **5.4** | **4.1** | **5.0** | **3.9** |
| **SD of litter size** | **3.2** | **0.0** | **2.2** | **2.5** | **2.5** | **2.1** | **2.1** | **2.0** |
| **% Died in 1st three days of life** | **5.1%** | **5.6%** | **15.8%** | **13.6%** | **42.4%** | **36.0%** | **16.8%** | **36%** |
| **% of total that died by**  **weaning ~4 weeks** | **5.9%** | **11.1%** | **23.0%** | **32.2%** | **59.3%** | **44.0%** | **20.0%** | **42%** |
| **Avg Length of Mating till 1st**  **Litter:** | **29** | **22** | **34** | **25** | **35** | **28** | **26** | **34** |
| **Total number of unique**  **mating males:** | **7** | **1** | **12** | **5** | **6** | **7** | **13** | **16** |
| **Total number of mating pairs**  **set up:** | **8** | **1** | **>16** | **14** | **10** | **11** | **22** | **17** |
| **Total number of mating pairs with progeny** | **7** | **1** | **N/A** | **7** | **4** | **11** | **21** | **15** |
| **% females who became pregnant and gave birth at**  **least once:** | **87.5%** | **100.0%** | **N/A** | **50.0%** | **40.0%** | **100.0%** | **95.5%** | **88.2%** |

Supplementary File 1e. Genotypes of offspring from *Naa12^+/-^* female mice crossed to the *Naa12^+/-^* male mice.

| **Genotype**  **(Expected Mendelian %)** | **Naa12^+/+^**  **(25%)** | **Naa12^+/-^**  **(50%)** | **Naa12^-/-^**  **(25%)** |
| --- | --- | --- | --- |
| **Adults (n=117)** | 26 (22%) | 62 (53%) | 29 (25%) |

Expected and observed Mendelian ratio of genotypes in offspring from crosses.

Supplementary File 1f. Genotypes of offspring from *Naa10^+/-^* *Naa12^+/+^* female mice crossed to the *Naa10^+/y^ Naa12^+/-^* male mice.

| **Genotype**  **(Expected Mendelian %)** | ***Naa10^(+/y)^* *Naa12^(+/-)^* males**  **(12.5%)** | ***Naa10^(-/y)^***  ***Naa12^(+/-)^* males**  **(12.5%)** | ***Naa10^(+/y)^* *Naa12^(+/+)^* males**  **(12.5%)** | ***Naa10^(-/y)^ Naa12^(+/+)^* males**  **(12.5%)** |
| --- | --- | --- | --- | --- |
| **Newborn pups (n=214)** | 27 (12.6%) | 0 (0%) | 33 (15.4%) | 21 (9.8%) |
| **Genotype**  **(Expected Mendelian %)** | ***Naa10^(+/-)^* *Naa12^(+/+)^* females**  **(12.5%)** | ***Naa10^(+/+)^***  ***Naa12^(+/+)^* females**  **(12.5%)** | ***Naa10^(+/-)^***  ***Naa12^(+/-)^* females**  **(12.5%)** | ***Naa10^(+/+)^* *Naa12^(+/-)^* females**  **(12.5%)** |
|  | 31 (14.5%) | 33 (15.4%) | 9 (4.2%) | 41 (19.2%) |
| **Early neonatal death, unable to genotype = 19 (8.9%)** | | | | |

Expected and observed Mendelian ratio of genotypes in offspring from crosses.

Supplementary File 1g. Genotypes of offspring from *Naa10^+/-^* *Naa12^+/+^* female mice crossed to the *Naa10^+/y^ Naa12^-/-^* male mice.

| **Genotype**  **(Expected Mendelian %)** | **NAA10^+/Y^ NAA12^+/-^**  **male (25%)** | **NAA10^-/Y^ NAA12^+/-^**  **male (25%)** | **NAA10^+/+^ NAA12^+/-^**  **female (25%)** | **NAA10^+/-^ NAA12^+/-^female (25%)** |
| --- | --- | --- | --- | --- |
| **Newborn pups (n=252*)** | 78 (31%) | 0 (0%) | 83 (33%) | 36 (14%) |
| ***Early neonatal death, unable to genotype = 55 (22%)** | | | | |

Expected and observed Mendelian ratio of genotypes in offspring from crosses.

Supplementary File 1h. Mendelian and Observed Offspring Distributions from Naa10(+/Y); Naa12(+/-) Male and Naa10(+/-); Naa12(+/-) Female Breeding

|  | | **Mendelian Genotype Distribution (%)** | | | **Observed Number (% Genotyped)** | | |
| --- | --- | --- | --- | --- | --- | --- | --- |
| **#*** | **Offspring Genotypes** | **F** | **M** | **Total** | **E10.5** | **E18.5** | **Postnatal** |
| 1 | Naa10 (+/+), Naa12 (+/+) | 6.25 |  | 6.25 | 2 (6.9) | 5 (15.2) | 16 (10.2) |
| 2 | Naa10 (+/+), Naa12 (+/-) | 12.50 |  | 12.50 | 4 (13.8) | 7 (21.2) | 28 (17.8) |
| 3 | Naa10 (+/+), Naa12 (-/-) | 6.25 |  | 6.25 | 3 (10.3) | 1 (3.0) | 16 (10.2) |
| 4 | Naa10 (+/-), Naa12 (+/+) | 6.25 |  | 6.25 | 3 (10.3) | 1 (3.0) | 14 (8.9) |
| 5 | Naa10 (+/-), Naa12 (+/-) | 12.50 |  | 12.50 | 6 (20.7) | 5 (15.2) | 5 (3.2) |
| 6 | Naa10 (+/-), Naa12 (-/-) | 6.25 |  | 6.25 | 2 (6.9) | 0 (0.0) | 0 (0.0) |
| 7 | Naa10 (+/Y), Naa12 (+/+) |  | 6.25 | 6.25 | 1 (3.4) | 4 (12.1) | 17 (10.8) |
| 8 | Naa10 (+/Y), Naa12 (+/-) |  | 12.50 | 12.50 | 1 (3.4) | 5 (15.2) | 31 (19.7) |
| 9 | Naa10 (+/Y), Naa12 (-/-) |  | 6.25 | 6.25 | 3 (10.3) | 3 (9.1) | 17 (10.8) |
| 10 | Naa10 (-/Y), Naa12 (+/+) |  | 6.25 | 6.25 | 0 (0.0) | 1 (3.0) | 17 (8.3) |
| 11 | Naa10 (-/Y), Naa12 (+/-) |  | 12.50 | 12.50 | 4 (13.8) | 1 (3.0) | 0 (0.0) |
| 12 | Naa10 (-/Y), Naa12 (-/-) |  | 6.25 | 6.25 | 0 (0.0) | 0 (0.0) | 0 (0.0) |
|  | **TOTAL (% Genotyped)** | **50.0** | **50.0** | **100.0** | **29 (99.8)** | **33 (100.0)** | **157 (99.9)** |
|  | **Not Genotyped (% Total)** |  |  |  | **3 (9.4)** | **23 (41.1)** | **0 (0.0)** |
|  | **TOTAL** | **50.0** | **50.0** | **100.0** | **32** | **56** | **157** |

F = Female; M = Male
*Genotypes in subsequent tables are numbered according to this table, which includes all possible genotypes from all crosses considered.

Supplementary File 1i. Mendelian and Observed Offspring Distributions from Naa10(+/Y); Naa12(-/-) Male and Naa10(+/-); Naa12(+/-) Female Breeding

|  | | **Mendelian Genotype Distribution (%)** | | | **Observed Number (% Genotyped)** | | | | |
| --- | --- | --- | --- | --- | --- | --- | --- | --- | --- |
| **#*** | **Offspring Genotypes** | **F** | **M** | **Total** | **E8.5** | **E10.5** | **E12.5** | **E18.5** | **Postnatal** |
| 2 | Naa10 (+/+), Naa12 (+/-) | 12.5 |  | 12.5 | 4 (19.0) | 3 (10.7) | 4 (16.0) | 2 (18.2) | 45 (25.1) |
| 3 | Naa10 (+/+), Naa12 (-/-) | 12.5 |  | 12.5 | 6 (28.6) | 8 (28.6) | 1 (4.0) | 2 (18.2) | 35 (19.6) |
| 5 | Naa10 (+/-), Naa12 (+/-) | 12.5 |  | 12.5 | 2 (9.5) | 8 (28.6) | 7 (28.0) | 3 (27.3) | 12 (6.7) |
| 6 | Naa10 (+/-), Naa12 (-/-) | 12.5 |  | 12.5 | 1 (4.8) | 1 (3.6) | 0 (0.0) | 0 (0.0) | 0 (0.0) |
| 8 | Naa10 (+/Y), Naa12 (+/-) |  | 12.5 | 12.5 | 1 (4.8) | 3 (10.7) | 7 (28.0) | 0 (0.0) | 40 (22.3) |
| 9 | Naa10 (+/Y), Naa12 (-/-) |  | 12.5 | 12.5 | 7 (33.3) | 4 (14.3) | 6 (24.0) | 4 (36.4) | 47 (26.3) |
| 11 | Naa10 (-/Y), Naa12 (+/-) |  | 12.5 | 12.5 | 0 (0.0) | 1 (3.6) | 0 (0.0) | 0 (0.0) | 0 (0.0) |
| 12 | Naa10 (-/Y), Naa12 (-/-) |  | 12.5 | 12.5 | 0 (0.0) | 0 (0.0) | 0 (0.0) | 0 (0.0) | 0 (0.0) |
| **TOTAL (% Genotyped)** | | **50.0** | **50.0** | **100.0** | **21 (100.0)** | **28 (100.1)** | **25 (100.0)** | **11 (100.1)** | **179 (100.0)** |
| **Not Genotyped (% Total)** | |  |  |  | **4 (16.0)** | **12 (30.0)** | **19 (43.2)** | **7 (38.9)** | **2 (1.1)** |
| **TOTAL** | | **50.0** | **50.0** | **100.0** | **25** | **40** | **44** | **18** | **181** |

F = Female; M = Male
*Genotypes in this table are numbered according to **Supplement File 1h**, which includes all possible genotypes from all crosses considered.

Supplementary File 1j. Mendelian and Observed Postnatal Offspring Distributions from Naa10(+/Y); Naa12(+/-) Male and Naa10(+/-); Naa12(+/+) Female Breeding

|  | | **Mendelian Genotype Distribution (%)** | | | **Observed Number (% Genotyped)** |
| --- | --- | --- | --- | --- | --- |
| **#*** | **Offspring Genotypes** | **F** | **M** | **Total** | **Postnatal** |
| 1 | Naa10 (+/+), Naa12 (+/+) | 12.5 |  | 12.5 | 33 (16.9) |
| 2 | Naa10 (+/+), Naa12 (+/-) | 12.5 |  | 12.5 | 41 (21.0) |
| 4 | Naa10 (+/-), Naa12 (+/+) | 12.5 |  | 12.5 | 31 (15.9) |
| 5 | Naa10 (+/-), Naa12 (+/-) | 12.5 |  | 12.5 | 9 (4.6) |
| 7 | Naa10 (+/Y), Naa12 (+/+) |  | 12.5 | 12.5 | 33 (16.9) |
| 8 | Naa10 (+/Y), Naa12 (+/-) |  | 12.5 | 12.5 | 27 (13.8) |
| 10 | Naa10 (-/Y), Naa12 (+/+) |  | 12.5 | 12.5 | 21 (10.8) |
| 11 | Naa10 (-/Y), Naa12 (+/-) |  | 12.5 | 12.5 | 0 (0.0) |
| **Total (% Genotyped)** | |  |  |  | **195 (99.9)** |
| **Not Genotyped (% Total)** | |  |  |  | **19 (8.9)** |
| **TOTAL** | | **50.0** | **50.0** | **100.0** | **214** |

F = Female; M = Male
*Genotypes in this table are numbered according to **Supplement File 1h**, which includes all possible genotypes from all crosses considered.

Supplementary File 1k. Mendelian and Observed Postnatal Offspring Distributions from Naa10(+/Y); Naa12(-/-) Male and Naa10(+/-); Naa12(+/+) Female Breeding

|  | | **Mendelian Genotype Distribution (%)** | | | **Observed Number (% Genotyped)** |
| --- | --- | --- | --- | --- | --- |
| **#*** | **Offspring Genotypes** | **F** | **M** | **Total** | **Postnatal** |
| 2 | Naa10 (+/+), Naa12 (+/-) | 25.0 |  | 25.0 | 83 (42.1) |
| 5 | Naa10 (+/-), Naa12 (+/-) | 25.0 |  | 25.0 | 36 (18.3) |
| 8 | Naa10 (+/Y), Naa12 (+/-) |  | 25.0 | 25.0 | 78 (39.6) |
| 11 | Naa10 (-/Y), Naa12 (+/-) |  | 25.0 | 25.0 | 0 (0.0) |
| **Total (% Genotyped)** | |  |  |  | **197 (100.0)** |
| **Not Genotyped (% Total)** | |  |  |  | **55 (21.8)** |
| **TOTAL** | | **50.0** | **50.0** | **100.0** | **252** |

F = Female; M = Male
*Genotypes in this table are numbered according to **Supplement File 1h**, which includes all possible genotypes from all crosses considered.

Supplementary File 1l. Mendelian and Observed Age-Specific Offspring Distributions from Four Crosses

|  | | **Observed Number at Age (% Genotyped)** | | | | |
| --- | --- | --- | --- | --- | --- | --- |
| **#*** | **Offspring Genotypes** | **E8.5** | **E10.5** | **E12.5** | **E18.5** | **Postnatal** |
| 1 | Naa10 (+/+), Naa12 (+/+) |  | 2 (3.5) |  | 5 (11.4) | 16 (4.8) |
| 2 | Naa10 (+/+), Naa12 (+/-) | 4 (19.0) | 7 (12.3) | 4 (16.0) | 9 (20.5) | 73 (21.7) |
| 3 | Naa10 (+/+), Naa12 (-/-) | 6 (28.6) | 11 (19.3) | 1 (4.0) | 3 (6.8) | 51 (15.2) |
| 4 | Naa10 (+/-), Naa12 (+/+) |  | 3 (5.3) |  | 1 (2.3) | 14 (4.2) |
| 5 | Naa10 (+/-), Naa12 (+/-) | 2 (9.5) | 14 (24.6) | 7 (28.0) | 8 (18.2) | 17 (5.1) |
| 6 | Naa10 (+/-), Naa12 (-/-) | 1 (4.8) | 3 (5.3) | 0 (0.0) | 0 (0.0) | 0 (0.0) |
| 7 | Naa10 (+/Y), Naa12 (+/+) |  | 1 (1.8) |  | 4 (9.1) | 17 (5.1) |
| 8 | Naa10 (+/Y), Naa12 (+/-) | 1 (4.8) | 4 (7.0) | 7 (28.0) | 5 (11.4) | 71 (21.1) |
| 9 | Naa10 (+/Y), Naa12 (-/-) | 7 (33.3) | 7 (12.3) | 6 (24.0) | 7 (15.9) | 64 (19.0) |
| 10 | Naa10 (-/Y), Naa12 (+/+) |  | 0 (0.0) |  | 1 (2.3) | 13 (3.9) |
| 11 | Naa10 (-/Y), Naa12 (+/-) | 0 (0.0) | 5 (8.8) | 0 (0.0) | 1 (2.3) | 0 (0.0) |
| 12 | Naa10 (-/Y), Naa12 (-/-) | 0 (0.0) | 0 (0.0) | 0 (0.0) | 0 (0.0) | 0 (0.0) |
| **TOTAL (% Genotyped)** | | **21 (100.0)** | **57 (100.2)** | **25 (100.0)** | **44 (100.2)** | **336 (100.0)** |

F = Female; M = Male
*Genotypes in this table are numbered according to **Supplement File 1h**, which includes all possible genotypes from all crosses considered.

Supplementary File 1m. Mendelian and Observed Cumulative Offspring Distributions from All Four Crosses

|  | | **Cumulative Observed Number (% Genotyped)** | | | | |
| --- | --- | --- | --- | --- | --- | --- |
| **#*** | **Offspring Genotypes** | **E8.5** | **E10.5** | **E12.5** | **E18.5** | **Postnatal** |
| 1 | Naa10 (+/+), Naa12 (+/+) |  | 2 (2.6) | 2 (1.9) | 7 (4.8) | 23 (4.8) |
| 2 | Naa10 (+/+), Naa12 (+/-) | 4 (19.0) | 11 (14.1) | 15 (14.6) | 24 (16.3) | 97 (20.1) |
| 3 | Naa10 (+/+), Naa12 (-/-) | 6 (28.6) | 17 (21.8) | 18 (17.5) | 21 (14.3) | 72 (14.9) |
| 4 | Naa10 (+/-), Naa12 (+/+) |  | 3 (3.8) | 3 (2.9) | 4 (2.7) | 18 (3.7) |
| 5 | Naa10 (+/-), Naa12 (+/-) | 2 (9.5) | 16 (20.5) | 23 (22.3) | 31 (21.1) | 48 (9.9) |
| 6 | Naa10 (+/-), Naa12 (-/-) | 1 (4.8) | 4 (5.1) | 4 (3.9) | 4 (2.7) | 4 (0.8) |
| 7 | Naa10 (+/Y), Naa12 (+/+) |  | 1 (1.3) | 1 (1.0) | 5 (3.4) | 22 (4.6) |
| 8 | Naa10 (+/Y), Naa12 (+/-) | 1 (4.8) | 5 (6.4) | 12 (11.7) | 17 (11.6) | 88 (18.2) |
| 9 | Naa10 (+/Y), Naa12 (-/-) | 7 (33.3) | 14 (17.9) | 20 (19.4) | 21 (18.4) | 91 (18.8) |
| 10 | Naa10 (-/Y), Naa12 (+/+) |  | 0 (0.0) | 0 (0.0) | 1 (0.7) | 14 (2.9) |
| 11 | Naa10 (-/Y), Naa12 (+/-) | 0 (0.0) | 5 (6.4) | 5 (4.9) | 6 (4.1) | 6 (1.2) |
| 12 | Naa10 (-/Y), Naa12 (-/-) | 0 (0.0) | 0 (0.0) | 0 (0.0) | 0 (0.0) | 0 (0.0) |
| **TOTAL (% Genotyped)** | | **21 (100.0)** | **78 (99.9)** | **103 (100.0)** | **147 (100.2)** | **483 (99.9)** |

F = Female; M = Male
*Genotypes in this table are numbered according to **Supplement File 1h**, which includes all possible genotypes from all crosses considered.

Supplementary File 1n. Mice analyzed by weighing, according to genotype.

| **Females** | | | | | | |
| --- | --- | --- | --- | --- | --- | --- |
|  | | | **Naa12 status** | | | |
|  |  |  | **WT/WT** | **WT/KO** | **KO/KO** | **Total** |
| **Naa10 status** | **Pure C57BL/6J background**  **Naa10 mice** | **WT/WT** | **67** | **N/A** | **N/A** | **67** |
|  |  | **WT/KO** | **125** | **N/A** | **N/A** | **125** |
|  |  | **KO/KO** | **15** | **N/A** | **N/A** | **15** |
|  |  | **Subtotal** | **207** | **N/A** | **N/A** | **207** |
|  | **mixed genetic background Naa10 and Naa12 mice** | **WT/WT** | **32** | **82** | **10** | **124** |
|  |  | **WT/KO** | **35** | **23** | **0** | **58** |
|  |  | **KO/KO** | **0** | **0** | **0** | **0** |
|  |  | **Subtotal** | **67** | **105** | **10** | **182** |
|  | **Total** | | **274** | **105** | **10** | **389** |

| **Males** | | | | | | |
| --- | --- | --- | --- | --- | --- | --- |
|  | | | **Naa12 status** | | | |
|  |  |  | **WT/WT** | **WT/KO** | **KO/KO** | **Total** |
| **Naa10 status** | **Pure C57BL/6J background**  **Naa10 mice** | **WT** | **97** | **N/A** | **N/A** | **97** |
|  |  | **KO** | **70** | **N/A** | **N/A** | **70** |
|  |  | **Subtotal** | **167** | **N/A** | **N/A** | **167** |
|  | **mixed genetic background Naa10 and Naa12 mice** | **WT** | **44** | **63** | **11** | **118** |
|  |  | **KO** | **14** | **0** | **0** | **14** |
|  |  | **Subtotal** | **58** | **63** | **11** | **132** |
|  | **Total** | | **225** | **63** | **11** | **299** |

Supplementary File 1o. Effects of *Naa10* KO on growth rate of *Naa10* mice on pure genetic background.

|  | **C57BL/6J inbred females (N = 207)** | | | | | | | | | | | | | | | |
| --- | --- | --- | --- | --- | --- | --- | --- | --- | --- | --- | --- | --- | --- | --- | --- | --- |
|  | **Effect of age and age^2^** | | | | **Effect of Naa10 KO** | | | | **Effect of age and Naa10 KO** | | | | **Effect of age,**  **Naa10 KO,**  **and interaction** | | | |
|  | **Coeff.** | **SE** | **z** | **p > \|z\|** | **Coeff.** | **SE** | **z** | **p > \|z\|** | **Coeff.** | **SE** | **z** | **p > \|z\|** | **Coeff.** | **SE** | **z** | **p > \|z\|** |
| **Age in days** | **0.349** | **0.006** | **59.49** | **< 0.001** |  |  |  |  | **0.349** | **0.006** | **59.38** | **< 0.001** | **0.344** | **0.010** | **34.59** | **< 0.001** |
| **Age^2^** | **- 0.001** | **0.00003** | **- 40.59** | **< 0.001** |  |  |  |  | **- 0.001** | **0.00003** | **- 40.56** | **< 0.001** | **- 0.001** | **0.00005** | **- 24.53** | **< 0.001** |
| **Naa10 KO** |  |  |  |  | **- 2.92** | **0.847** | **- 3.45** | **0.001** | **- 0.252** | **0.248** | **- 1.01** | **ns** | **- 0.219** | **0.451** | **- 0.49** | **ns** |
| **Age x KO** |  |  |  |  |  |  |  |  |  |  |  |  | **0.009** | **0.012** | **0.75** | **ns** |
| **Age^2^ x KO** |  |  |  |  |  |  |  |  |  |  |  |  | **-0.00009** | **0.00007** | **-1.27** | **ns** |
| **(constant)** | **2.518** | **0.204** | **12.35** | **< 0.001** | **17.23** | **0.690** | **24.96** | **< 0.001** | **2.697** | **0.272** | **9.93** | **< 0.001** | **2.633** | **0.382** | **6.90** | **< 0.001** |
| **Wald *X*^2^ ^a^** | **6547.29, p < 0.0001** | | | | **11.93, p = 0.0006** | | | | **6552.24, p < .0001** | | | | **6611.63, p < 0.0001** | | | |

|  | **C57BL/6J inbred males (N = 167)** | | | | | | | | | | | | | | | |
| --- | --- | --- | --- | --- | --- | --- | --- | --- | --- | --- | --- | --- | --- | --- | --- | --- |
|  | **Effect of age and age^2^** | | | | **Effect of Naa10 KO** | | | | **Effect of age and Naa10 KO** | | | | **Effect of age,**  **Naa10 KO,**  **and interaction** | | | |
|  | **Coeff.** | **SE** | **z** | **p > \|z\|** | **Coeff.** | **SE** | **z** | **p > \|z\|** | **Coeff.** | **SE** | **z** | **p > \|z\|** | **Coeff.** | **SE** | **z** | **p > \|z\|** |
| **Age in days** | **0.454** | **0.008** | **60.16** | **< 0.001** |  |  |  |  | **0.454** | **0.007** | **62.20** | **< 0.001** | **0.467** | **0.009** | **51.68** | **< 0.001** |
| **Age^2^** | **- 0.002** | **0.00005** | **- 39.76** | **< 0.001** |  |  |  |  | **-0.002** | **0.00004** | **- 41.16** | **< 0.001** | **- 0.002** | **0.00005** | **- 34.53** | **< 0.001** |
| **Naa10 KO** |  |  |  |  | **- 4.721** | **1.040** | **- 4.54** | **< 0.001** | **-2.578** | **0.304** | **- 8.47** | **< 0.001** | **- 1.351** | **0.504** | **- 2.68** | **0.007** |
| **Age x KO** |  |  |  |  |  |  |  |  |  |  |  |  | **- 0.035** | **0.015** | **- 2.32** | **0.020** |
| **Age^2^ x KO** |  |  |  |  |  |  |  |  |  |  |  |  | **0.0001** | **0.00009** | **1.46** | **ns** |
| **(constant)** | **1.303** | **0.271** | **4.81** | **< 0.001** | **19.56** | **0.668** | **29.28** | **< 0.001** | **2.430** | **0.283** | **8.58** | **< 0.001** | **1.931** | **0.321** | **6.01** | **< 0.001** |
| **Wald *X*^2^ ^a^** | **7220.56, p < 0.0001** | | | | **20.60, p < 0.0001** | | | | **8007.09, p < 0.0001** | | | | **8185.75, p < 0.0001** | | | |

^a^ The Wald *X*^2^ is a measure of the overall goodness of fit of the complete model.

Supplementary File 1p. Effects of *Naa10* and *Naa12* Kos on growth rate on mixed genetic background

|  | **females (N = 182): effects of age and knockouts on weight** | | | | | | | | | | | | | | | | | | | |
| --- | --- | --- | --- | --- | --- | --- | --- | --- | --- | --- | --- | --- | --- | --- | --- | --- | --- | --- | --- | --- |
|  | **Effect of age and age^2^** | | | | **Effects of age**  **and Naa10 KO** | | | | **Effects of age**  **and Naa12 KO** | | | | **Effects of age,**  **Naa10 & Naa12 Kos** | | | | **Effects: age,**  **Naa10, Naa12, both Kos** | | | |
|  | **Coeff** | **SE** | **z** | **p > \|z\|** | **Coeff** | **SE** | **z** | **p > \|z\|** | **Coeff** | **SE** | **z** | **p > \|z\|** | **Coeff** | **SE** | **z** | **p > \|z\|** | **Coeff** | **SE** | **z** | **p > \|z\|** |
| **Age** | **.** | **0.011** | **43.10** | **< 0.001** | **0.489** | **0.011** | **45.02** | **< 0.001** | **0.489** | **0.011** | **43.25** | **< 0.001** | **0.491** | **0.011** | **46.16** | **< 0.001** | **0.492** | **0.011** | **46.49** | **< 0.001** |
| **Age^2^** | **-0.003** | **0.0001** | **-24.47** | **< 0.001** | **-0.003** | **0.0001** | **-25.58** | **< 0.001** | **-0.003** | **0.0001** | **-24.55** | **< 0.001** | **-0.003** | **0.0001** | **-26.21** | **< 0.001** | **-0.003** | **0.0001** | **-26.45** | **< 0.001** |
| **Naa10** |  |  |  |  | **-1.117** | **0.254** | **-4.40** | **< 0.001** |  |  |  |  | **-1.424** | **0.267** | **-5.33** | **< 0.001** | **-0.368** | **0.335** | **-1.10** | **ns** |
| **Naa12-**  **Het** |  |  |  |  |  |  |  |  | **-0.377** | **0.247** | **-1.53** | **ns** | **-0.789** | **0.255** | **-3.09** | **0.002** | **0.039** | **0.287** | **0.14** | **ns** |
| **Naa12-**  **Ho** |  |  |  |  |  |  |  |  | **-0.659** | **0.625** | **-1.05** | **ns** | **-1.376** | **0.621** | **-2.21** | **0.027** |  |  |  |  |
| **Naa10-Naa12-**  **Het** |  |  |  |  |  |  |  |  |  |  |  |  |  |  |  |  | **-2.143** | **0.510** | **-4.20** | **< 0.001** |
| **(constant)** | **0.616** | **0.264** | **2.33** | **0.020** | **0.966** | **0.267** | **3.61** | **< 0.001** | **0.825** | **0.291** | **2.83** | **0.005** | **1.508** | **0.307** | **4.91** | **< 0.001** | **0.879** | **0.307** | **2.86** | **0.004** |
| **Wald *X*^2^ ^a^** | **4692.14, p < 0.0001** | | | | **5116.03, p < 0.0001** | | | | **4752.14, p < 0.0001** | | | | **5396.11, p < 0.0001** | | | | **5461.46, p < 0.0001** | | | |

|  | **females (N = 182): effects of age and knockouts on growth rate** | | | | | | | | | | | | | | | |
| --- | --- | --- | --- | --- | --- | --- | --- | --- | --- | --- | --- | --- | --- | --- | --- | --- |
|  | **Effects of age, Naa10 KO, interaction with age** | | | | **Effects of age, Naa12 KO, interaction with age** | | | | **Effects of age, both Kos & interaction with age** | | | | **Effects: age, Kos & interaction with ea. Other** | | | |
|  | **Coeff** | **SE** | **z** | **p > \|z\|** | **Coeff** | **SE** | **z** | **p > \|z\|** | **Coeff** | **SE** | **z** | **p > \|z\|** | **Coeff** | **SE** | **z** | **p > \|z\|** |
| **Age** | **0.508** | **0.013** | **39.24** | **< 0.001** | **0.491** | **0.015** | **32.64** | **< 0.001** | **0.523** | **0.018** | **29.09** | **< 0.001** | **0.496** | **0.011** | **46.35** | **< 0.001** |
| **Age^2^** | **-0.003** | **0.0001** | **-22.76** | **< 0.001** | **-0.003** | **0.0002** | **-17.97** | **< 0.001** | **-0.003** | **0.0002** | **-16.71** | **< 0.001** | **-0.003** | **0.0001** | **-26.24** | **< 0.001** |
| **Naa10** | **0.109** | **0.547** | **0.20** | **ns** |  |  |  |  | **0.030** | **0.582** | **0.05** | **ns** | **-0.364** | **0.336** | **-1.08** | **ns** |
| **Age x Naa10** | **-0.059** | **0.023** | **-2.51** | **0.012** |  |  |  |  | **-0.068** | **0.025** | **-2.76** | **0.006** |  |  |  |  |
| **Age^2^ x Naa10** | **0.0006** | **0.0002** | **2.25** | **0.024** |  |  |  |  | **0.0006** | **0.0003** | **2.46** | **0.014** |  |  |  |  |
| **Naa12-Het** |  |  |  |  | **-0.175** | **0.547** | **-0.32** | **ns** | **-0.099** | **0.557** | **-0.18** | **ns** | **0.036** | **0.288** | **0.13** | **ns** |
| **Age x Naa12-Het** |  |  |  |  | **-0.005** | **0.023** | **-0.23** | **ns** | **-0.029** | **0.023** | **-1.23** | **ns** |  |  |  |  |
| **Age^2^ x Naa12-**  **Het** |  |  |  |  | **0.000004** | **0.0002** | **0.02** | **ns** | **0.0002** | **0.0002** | **0.97** | **ns** |  |  |  |  |
| **Naa12-Ho** |  |  |  |  | **-0.882** | **1.584** | **-0.56** | **ns** | **-0.916** | **1.551** | **-0.59** | **ns** |  |  |  |  |
| **Age x Naa12-Ho** |  |  |  |  | **0.003** | **0.073** | **0.04** | **ns** | **-0.029** | **0.071** | **-0.41** | **ns** |  |  |  |  |
| **Age^2^ x Naa12-Ho** |  |  |  |  | **0.00003** | **0.0007** | **0.04** | **ns** | **0.0003** | **0.001** | **0.47** | **ns** |  |  |  |  |
| **Naa10-Naa12-Het** |  |  |  |  |  |  |  |  |  |  |  |  | **0.784** | **1.303** | **0.60** | **ns** |
| **Age x**  **Naa10-Naa12-Het** |  |  |  |  |  |  |  |  |  |  |  |  | **-0.113** | **0.052** | **-2.15** | **0.031** |
| **Age^2^ x**  **Naa10-Naa12-Het** |  |  |  |  |  |  |  |  |  |  |  |  | **0.0009** | **0.0005** | **1.73** | **ns** |
| **(constant)** | **0.588** | **0.305** | **1.93** | **ns** | **0.756** | **0.348** | **2.17** | **0.030** | **0.790** | **0.426** | **1.85** | **ns** | **0.774** | **0.309** | **2.50** | **0.012** |
| **Wald *X*^2^ ^a^** | 5224.04, p < 0.0001 | | | | 4758.89, p < 0.0001 | | | | 5526.71, p < 0.0001 | | | | 5586.86, p < 0.0001 | | | |

Het : heterozygous
Ho : homozygous
^a^ The Wald *X*^2^ is a measure of the overall goodness of fit of the complete model.
